# 1st International Experts’ Meeting on Agitation: Conclusions Regarding the Current and Ideal Management Paradigm of Agitation

**DOI:** 10.3389/fpsyt.2018.00054

**Published:** 2018-02-27

**Authors:** José Martínez-Raga, Mario Amore, Guido Di Sciascio, Radu Ioan Florea, Marina Garriga, Gabriel Gonzalez, Kai G. Kahl, Per-Axel Karlsson, Jens Kuhn, Maria Margariti, Bruno Pacciardi, Konstantinos Papageorgiou, Maurizio Pompili, Fabrice Rivollier, Ángel Royuela, Gemma Safont, Joachim Scharfetter, Bo Skagen, Kazuhiro Tajima-Pozo, Pierre Vidailhet

**Affiliations:** ^1^Hospital Universitario Doctor Peset, University of Valencia & CEU Cardenal Herrera University, Valencia, Spain; ^2^San Martino Hospital, Genova, Italy; ^3^Azienda Ospedaliero Universitaria "Consorziale Policlinico" di Bari, Bari, Italy; ^4^Hôpitaux Universitaires de Montpellier, Montpellier, France; ^5^Hospital Clínic de Barcelona, Barcelona, Spain; ^6^Hospital de Emergencias Psiquiátricas Torcuato de Alvear, Buenos Aires, Argentina; ^7^Medizinische Hochschule, Hannover, Germany; ^8^Norrbottens Läns Landsting, Luleå, Sweden; ^9^Johanniter Krankenhaus, Evangelisches Klinikum Niederrhein, Oberhausen, Germany; ^10^Medical School of Athens, National and Kapodistrian University of Athens, Eginition Hospital, Athens, Greece; ^11^Azienda Ospedaliera Universitaria Pisana, Pisa, Italy; ^12^Medical University of Vienna, Vienna, Austria; ^13^II Medical School, Sapienza University of Rome, Rome, Italy; ^14^Centre Hospitalier Sainte Anne, Paris, France; ^15^Complejo asistencial Universitario de Palencia, Palencia, Spain; ^16^Hospital Universitari Mútua Terrassa, Universitat de Barcelona, Barcelona, Spain; ^17^Danube Hospital, Vienna, Austria; ^18^Øyane DPS Bergen, Bergen, Norway; ^19^Hospital Universitario Fundación Alcorcón, Alcorcón, Spain; ^20^Hôpitaux Universitaires de Strasbourg, Strasbourg, France

**Keywords:** schizophrenia, bipolar disorder, psychomotor agitation, management, clinical practice

## Abstract

Agitation is a heterogeneous concept without a uniformly accepted definition, however, it is generally considered as a state of cognitive and motor hyperactivity characterized by excessive or inappropriate motor or verbal activity with marked emotional arousal. Not only the definition but also other aspects of agitated patients’ care are still unsolved and need consensus and improvement. To help the discussion about agitation among experts and improve the identification, management, and treatment of agitation, the 1st International Experts’ Meeting on Agitation was held in October 2016 in Madrid. It was attended by 20 experts from Europe and Latin America with broad experience in the clinical management of agitated patients. The present document summarizes the key conclusions of this meeting and highlights the need for an updated protocol of agitation management and treatment, the promotion of education and training among healthcare professionals to improve the care of these patients and the necessity to generate clinical data of agitated episodes.

## Introduction

Patients with acute psychomotor agitation are commonly attended in medical and psychiatric clinical settings. Agitation is a heterogeneous concept without a uniformly accepted definition; however, it is generally considered as a state of cognitive and motor hyperactivity characterized by excessive or inappropriate motor or verbal activity with marked emotional arousal (1–3). While it is difficult to estimate the prevalence of acute agitation episodes due to the scarcity of epidemiological studies, it is widely recognized that agitation is a common phenomenon, both in medical and psychiatric emergency and inpatient settings. Acute episodes of psychomotor agitation account for 900,000 annual visits to psychiatric emergency services (4) and represent a total of 1.7 million emergency department visits per year in the USA (5). Furthermore, it is estimated that up to 10% of psychiatric emergencies attended at emergency services involve an acute agitation episode (6–8). Agitation is frequently associated with an underlying psychiatric disorder, particularly schizophrenia, bipolar disorder, personality disorders, Alzheimer’s disease, or other types of dementia, among others (6, 9, 10). In cases of dementia, there are multiple factors that can precipitate or worsen psychomotor agitation, such as medical problems, drug adverse events, sleep abnormalities, delirium, depression, or environmental and social factors, but it is mostly linked to the severity of the neurocognitive disorder (11). Treatment of the agitated patient is complex, due to the status of the patient, the involvement of multiple healthcare professionals (HCPs), and the ethical/legal concerns on the different management procedures.

Various guidelines are available providing guidance on the medical and psychiatric assessment and the pharmacological and non-pharmacological management of the patient presenting with an episode of agitation, including the recent guidelines from Austrian Society for Neuro-Psychopharmacology and Biological Psychiatry (12), the recommendations from the World Federation of Societies of Biological Psychiatry (WFSBP) regarding management of acute agitation in schizophrenia (13), the Project BETA (Best practices in Evaluation and Treatment of Agitation) guidelines from the American Association for Emergency Psychiatry (2, 14–17), the Clinical Policy for Diagnosis and Management of the Adult Psychiatric Patient in the Emergency Department from the American College of Emergency Physicians’ (18), the guideline for Violence: the short-term management of disturbed/violent behavior in inpatient settings and emergency departments—CG25—issued by the United Kingdom National Institute for Health and Care Excellence (NICE) in 2005 (19) or the standards in Restraint and Seclusion proposed by the Joint Commission on Accreditation of Healthcare Organizations and the Centers for Medicare and Medicaid (20). In addition, the Expert consensus paper on the assessment management of agitation in psychiatry from the WFSBP has recently compiled the state-of-art of the assessment and management of agitation (21). In spite of all these guidelines, care of the agitated patient remains often suboptimal, partly as a result of the failure to implement updated consensus recommendations and treatment algorithms, and partly due to the heterogeneous etiology and complexity of the clinical presentation of the episode of agitation. An agreement for clearer patient-centered protocols that can be followed by all HCPs involved in the management of agitated patients in any clinical setting is an important unmet need.

A group of 20 experts from nine different countries across Europe and South America with an interest and large experience in the clinical management of agitated patients met in Madrid for the 1st International Experts’ Meeting on Agitation. This independent expert panel was set up to identify the difficulties and barriers HCPs face in daily clinical practice in the assessment and management of agitated patients in emergency and inpatient settings and to discuss practical recommendations to best improve them. Timely evaluation of precipitating factors and a prompt intervention are crucial to prevent escalation to violent behaviors and potentially dangerous situations. The objectives of the expert meeting and the present document is to increase awareness on the adequate assessment, early identification and on the optimal and most efficient individualized therapeutic interventions of an episode of agitation. The key topics addressed in this statement are as follows: (1) definition and identification of agitation, (2) current management practices of an episode of agitation, (3) ideal treatment interventions, and (4) current barriers and potential solutions for better management and treatment practices of agitated patients.

## Methods

The present document is the result of a full-day meeting held in October 2016 in Madrid in which the need for improved communication and a practical consensus statement were identified. The meeting was attended by 20 Psychiatry experts actively involved in the clinical management of agitated patients from Argentina, Austria, Finland, France, Germany, Greece, Italy, Norway, Spain, and Sweden. The meeting was organized in different sections, where participants had the opportunity to discuss different concepts in groups using Workmats^®^ (22) and then shared the results with the rest of the groups to start a debate and reach conclusions. Workmat^®^ methodology consists in exercises presented in a poster format designed to maximize the interaction among participants and sharing of ideas to reach agreements and conclusions on a specific subject (22). The present document that has been reviewed and discussed within the expert group is the result of the debates during the meeting and the exchange of information and proposals prior and after the meeting. Following this process, the final document was circulated for written approval by all members of the Expert Group. For the preparation of the document, non-systematic search for clinical studies, randomized controlled trials, and reviews was performed from Medline, as well as from cross-referencing and identification by the participating experts. Details of the clinical presentation, assessment, and management of agitated patients were further informed by clinical expertise. Results and conclusions shown here represent the general views agreed by the experts group for episodes of agitation. Regarding the treatment, the discussion focused primarily on the pharmacological options. This practical consensus statement is written for specialists in psychiatry but is also intended to increase the understanding of acute agitation episodes from other healthcare providers.

## Key Results and Conclusion

### Definition and Identification of Psychomotor Agitation

There is no consensus on the definition of agitation and on what elements should be included in this clinical entity. Moreover, the identification of agitation and the assessment of potential escalation of symptoms are still a matter of controversy. Therefore, the first exercise during the expert meeting was to develop a definition of agitation that could allow specialized and non-specialized HCPs to identify an agitation episode at an early stage. The expert discussion consisted in choosing, from an open list, the signs and/or symptoms that would better help identify psychomotor agitation to specialized and non-specialized HCPs.

The group concluded that alterations in physical parameters may be useful but they have limited relevance in differentiating agitation from other pathological states. Cognitive alterations have also limited practical use. By contrast, behavioral symptoms were found to be relevant in order to quickly identify episodes of agitation and even though further assessment may provide a definitive diagnosis, the following four signs were identified as helpful to provide a preliminary identification of agitation:

1. Inability to stay calm or still,
2. Motor and verbal hyperactivity and hyperresponsiveness,
3. Emotional tension,
4. Difficulties in communication.

Following the discussion during this initial exercise, agitation was defined as “A state where patients cannot remain still or calm, characterized by internal features such as hyperresponsiveness, racing thoughts and emotional tension; and external ones, mainly motor and verbal hyperactivity, and communication impairment.” It was recognized that while this definition may not be the most inclusive and accurate, it has a practical component that may sacrifice some scientific precision for increased applicability. However, the identification process relays, at least partly, on the experience of the HCPs facing the patient and the general feeling that the patient is agitated. This is a work-in-progress definition that needs to be validated due to the complexity of agitation and the many different levels of severity and presentations that can be found.

### Management of Psychomotor Agitation

**Figure 1:**
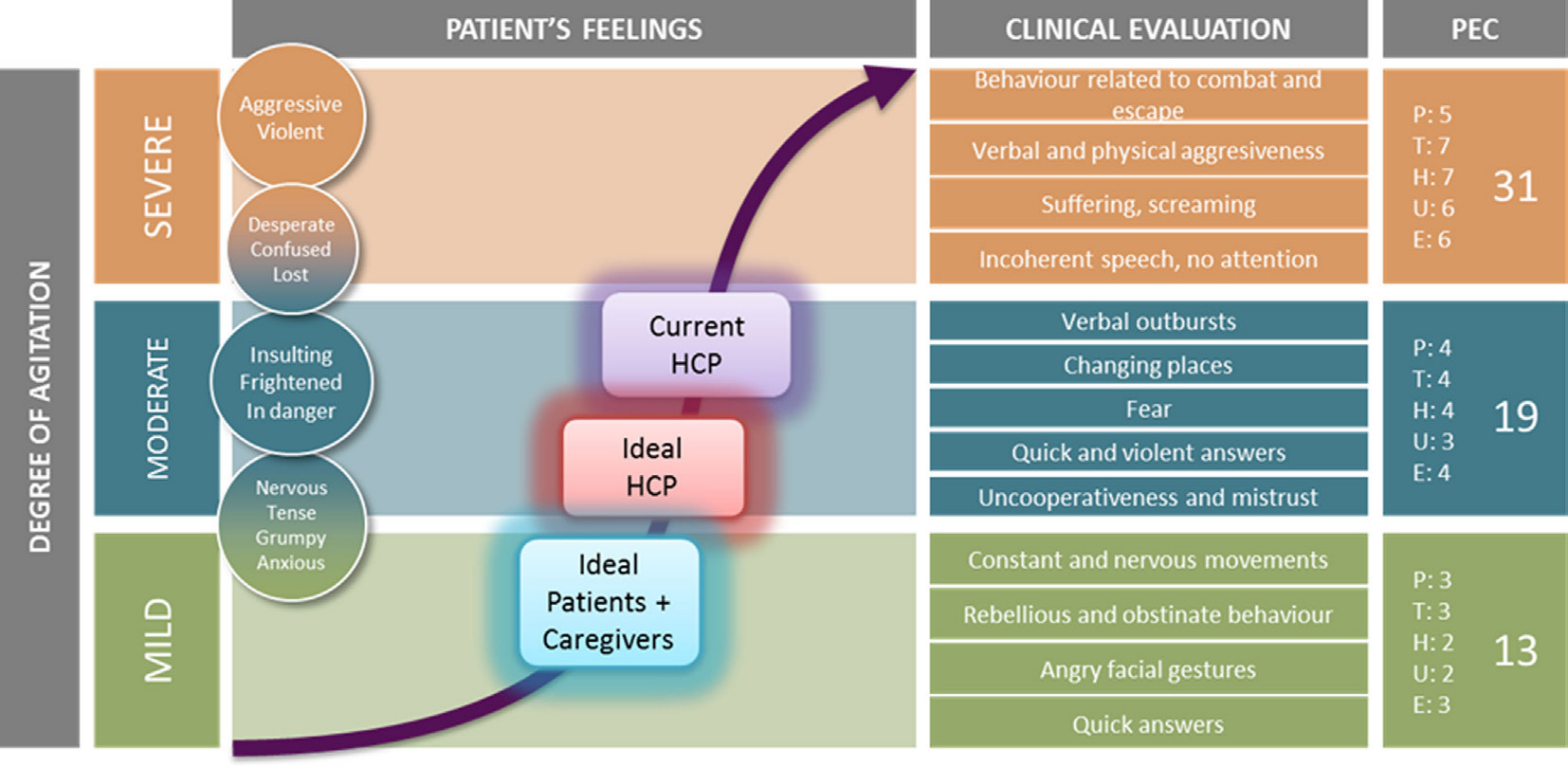
Agreed current and ideal acting-point for healthcare professional (HCPs) and patients/caregivers.

Agitation is a dynamic situation that may rapidly escalate from mild (distress, restless, worry, fear, etc.) to loss of control (violence, aggressiveness, confusion, etc.) and different scales have been developed to measure the severity of agitation (2, 21, 23–25). Given the continuum clinical presentation of agitation, it may be often difficult to identify the best moment for intervening in order to optimize the patients’ outcome. During this discussion process, experts had to define both: their current acting-point during an episode of agitation and the ideal acting-point (for HCPs and also for patients) based on a curve representing time in the *X* axis and the Positive and Negative Syndrome Scale-Excited Component (26) in the *Y* axis (Figure 1).

The group agreed that intervention aimed at avoiding the “point of no return,” where escalation cannot be controlled only by non-pharmacological interventions, but the exact moment was controversial. The current acting-point in clinical practice for the panel participants was right before reaching levels of a severe degree of agitation. In addition, it was acknowledged that pharmacological treatment is usually avoided or postponed in the presence of mild stages of agitation because (I) the episode may de-escalate without further treatment, (II) patients or their caregivers prefer avoiding medication or (III) non-pharmacological treatments, such as verbal de-escalation, are considered the first-choice at that stage. During the discussion, the experts agreed that it would be beneficial to intervene as early as possible, between the low-moderate to mid-moderate level of severity. This would reduce the chances of escalation and would improve the patient cooperativeness and final clinical outcome. Moreover, when experts considered the preference of patients and caregivers, the gap was even larger, as the preferred ideal intervention point was situated in the mild levels of severity (Figure 1). To improve the patient experience and avoid escalation of symptoms, intervention should be considered earlier, probably before reaching moderate levels of agitation. This would improve patient cooperation, and consequently would allow using less invasive/traumatic treatments. The benefits of an early intervention are clearly superior to the risks (Table 1), thus earlier intervention was strongly recommended.

**Table 1:** Benefits and risks of an earlier intervention during an episode of agitation.

Benefits	Broader set of intervention choices, both pharmacological and non-pharmacological
	Patient cooperation
	Less traumatic/invasive treatments
	Increased therapeutic alliance
	Potentially, reduced waiting time until a full diagnosis can be made
	Reduction of medical and economic resources needed

Drawbacks	Agitation misidentification
	Safety issues due to the use of drugs that could have been avoided

### Pharmacological Treatment of Psychomotor Agitation

Due to the diversity of underlying causes of agitation and the different degrees of severity, choice of best treatment option is not always easy. In general, it was acknowledged by the expert group that there are two main trends to treat agitated patients. (I) “Imposing treatment,” which tends to occur when the psychiatrist is not familiar with the patient (seen for the first time) or in episodes of severe agitation. It includes more coercive interventions and invasive treatments such as intramuscular medications and (II) “Proposing treatment,” which occurs more commonly when the psychiatrist is familiar with the patient or in cases of mild agitation. It includes less intrusive management, such as verbal de-escalation, usually leading to patient cooperation and, if pharmacological treatment is needed, less invasive options can be proposed to the patient, such as oral or inhaled medications. During one of the exercises, the participants were asked to state the approximate frequency of use of the different pharmacological options. Despite the fact that there was a large variety of choices depending on the different countries, or even depending on the various clinical settings, in general, antipsychotics (APs) were the most frequently used (more than 50%); followed by benzodiazepines (BZD), and just a few participants used other choices such as anti-depressants, anti-histamine medications, so on. Within the APs, oral and intramuscular formulations were currently the preferred ones, leaving the other formulations as second choices. Inhaled APs were still not commonly used, although there was a trend toward increasing their use.

During the session, experts focused on the characteristics of a hypothetical ideal pharmacological treatment for agitation and, despite the differences in available pharmacological options in each setting, which of the current available treatments were positioned closest to the ideal. The different characteristics, described in the consensus (21), were presented to the participants and they had to score each route of administration from 1 to 10 (with 10 being closest to the ideal treatment) based on available data from studies in the literature and on the perception and experience of the participants. The characteristics evaluated were as follows:

- Rapid onset of action,
- Calmness without sedation,
- Easy to administer,
- Non-invasive,
- Non-traumatic/non-coercive,
- Safety profile,
- Favorable tolerability,
- Patient preference,
- Promote long-term adherence to treatment of the underlying condition.

**Figure 2:**
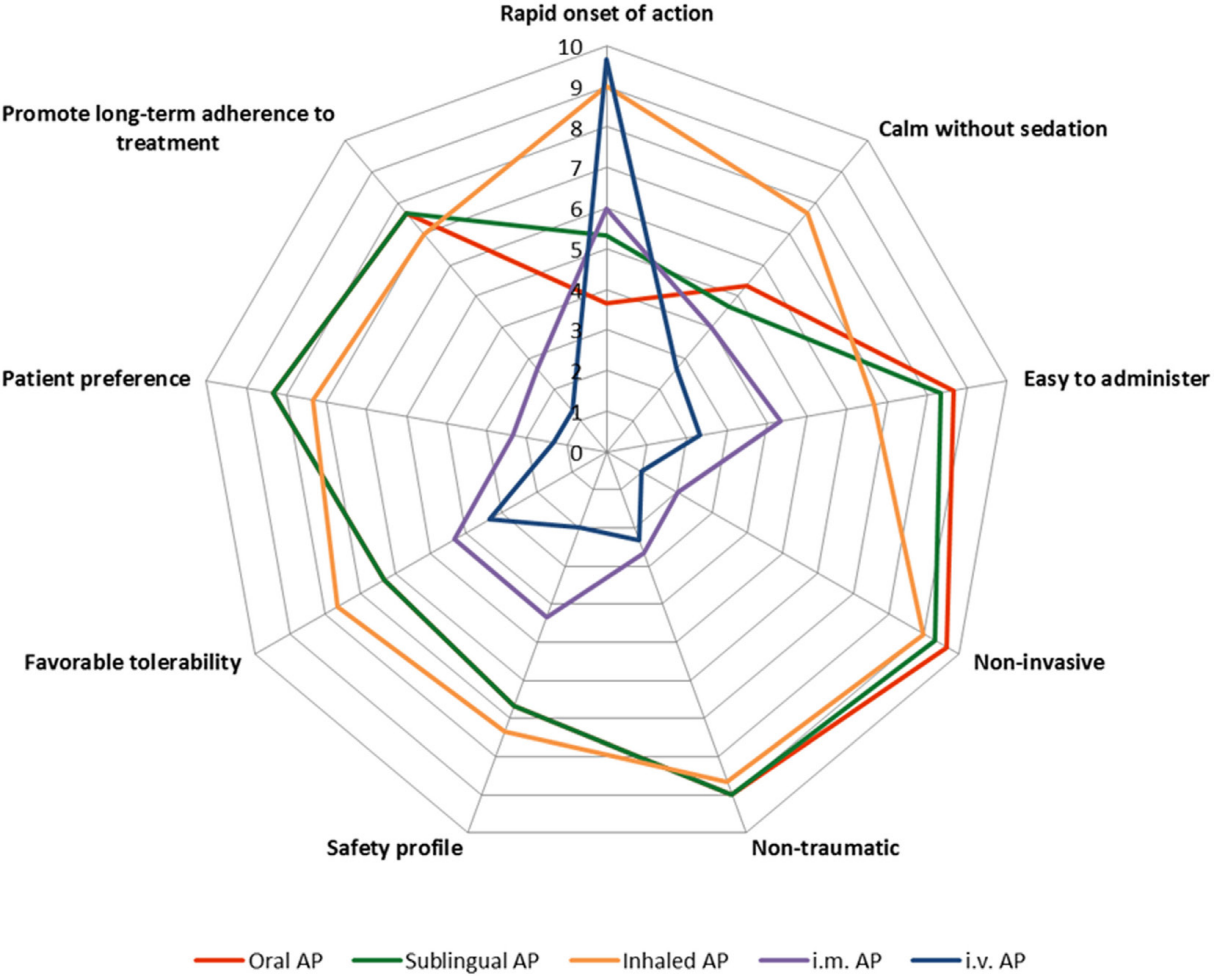
Spidermap of different antipsychotic (AP) formulations based on the average score for each one of the different characteristics, as resulted from the session.

Interestingly, three different treatment groups emerged based on the overall characteristics profile (Figure 2):

- Intramuscular and intravenous AP formulations: both routes of administration were acknowledged to have similar profiles, scoring high in rapidness of action but relatively low in the rest of therapeutic properties. This was of particular relevance for the patient, due to their coercive and invasive nature.
- Oral and sublingual AP formulations: Both routes of administration scored very similar for all the features, with high scores for ease of administration, non-invasive, and non-traumatic characteristics but scoring below average for rapidness of action and in calming without sedation.
- Inhaled AP formulation: this represents a different treatment option based on the characteristic profile, with very rapid onset of action and calming without sedating as the key features. Inhaled AP were identified as having combined positive features of the two other groups, similar rapidness as the intramuscular and intravenous formulations, and similar non-invasive/traumatic, safety/tolerability, and patient preference characteristics to the oral and sublingual formulations. In addition, regarding the capacity of calming without sedating, the inhaled AP formulation was rated significantly higher than the rest.

During the discussion, all the therapeutic characteristics were considered as having similar weight. Consequently, there was limited debate on which of the different features were more relevant for the selection of a pharmacological treatment of agitation. There was also some discussion on whether treatment decisions should be made based only on a limited number of characteristics. During the debate, a rapid onset of action, calming without sedating and convenience (including ease to administer, non-invasive/traumatic) were the features that occupied more time during the discussion.

In conclusion, according to this exercise, inhaled APs have characteristics that would be more similar to an ideal treatment, with a rapid onset of action, calming without sedating effects and being non-invasive/traumatic. However, as identified by expert participants, use of these inhaled APs in daily clinical practice remains very low compared to other formulations, partly due to limited experience, knowledge of the product or inertia from previous practices. One of the potential limitations of these conclusions was that the exercise only evaluated individual treatments and did not consider potential combinations of different medications.

### Barriers and Solutions for the Effective Management and Treatment of Agitation

**Figure 3:**
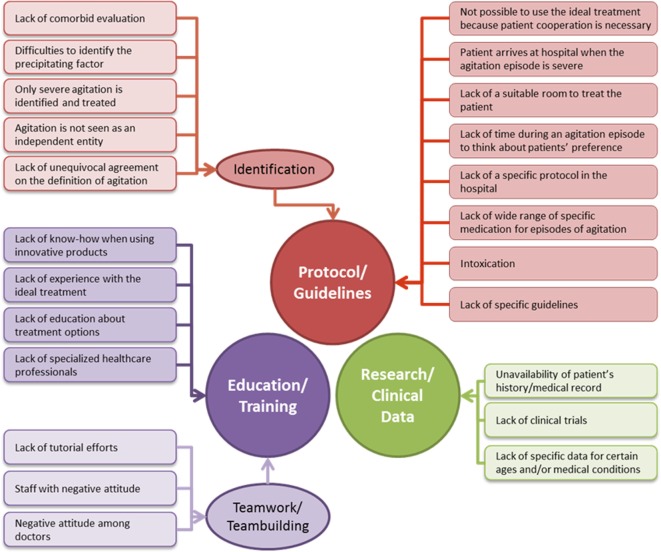
Main barriers for the improvement in the management and treatment of agitation grouped by category.

During the session, experts were able to discuss the most relevant barriers to reach optimal procedures in the care of the acute agitated patient and provide solutions to improve the current management. Some barriers were presented to participants and they could add further suggestions as needed. It became clear that most of the barriers were linked and interconnected to each other. Three main categories were identified as the main barriers to improve agitation care (Figure 3): Lack of protocol/guidelines (including guidelines to identify agitation), below optimal education/training, and limited clinical data. In parallel with the barriers, the solutions that were suggested could also be grouped under the same three categories.

#### Elaboration of a Protocol/Guideline for Agitation

Some of the barriers were related to the difficulty of identification, the know-how, or the lack of a clear decision-making algorithm. Most of the experts agreed that the elaboration of an updated management/treatment algorithm would be a high priority; this would help state clear indications for specialists and other HCPs in terms of identification, management, and treatment. Furthermore, this would also help to standardize procedures, moving away from deeply in rooted suboptimal practices, and to train clinicians in emergency room and hospital medical teams. The specific benefits would include an earlier intervention, use of closer-to-ideal treatments, improvement of therapeutic alliance, and optimization of hospital resources.

#### Education/Training

Another set of the key barriers were related to the lack of specific training programs for HCPs and teams for the management and treatment of agitation. Two components were acknowledged as necessary to improve agitation outcomes: (I) a basic educational program for HCPs involved in the management of agitation, aiming to improve the abilities in identifying (symptoms, level of severity, etc.) and on how to approach agitation (non-pharmacological treatments, APs, BZDs, characteristics of the different formulations, physical restraint when needed, etc.), based on current practices and clinical and evidence-based data, and (II) improved training programs to prepare for the practical situations of dealing with an agitated patient in the specific settings, which should include training on verbal de-escalation, strategies to improve the therapeutic alliance, such as intervening earlier, more rapidly and in a respectful/ethical manner, practical lessons on the use of available treatment tools, etc. It was agreed that it is very important to conduct the training in a collaborative environment to reach a multidisciplinary approach and improve coordinated and collaborative teamwork.

#### Research/Clinical Data

Evidence-based clinical data are published continuously. Therefore, frequent review of the literature to update guidelines and safety information was acknowledged as being highly important. In addition, clinical data from each hospital database/registry are also relevant to understand how efficient the management and treatment of agitation is. Without proper record forms of patients with agitation (electronic format is highly recommended), there will be a lack of real-world evidence to make decisions and progressively optimize and update guidelines locally. The expert group agreed as well that clinical data (from clinical trials or from centers’ databases) are also relevant to address issues related to comorbidities or other patient characteristics (e.g., age, gender, medical conditions, and so on), which would allow a more personalized treatment protocol.

## Discussion

Episodes of psychomotor agitation represent a significant challenge in the emergency department and inpatient settings, both for non-psychiatric and psychiatric clinical staff, as well as for patients and caregivers. Furthermore, agitation and most of the interventions and treatment strategies have consequences beyond the actual episode. Management of agitation has been associated with greater use of healthcare resources, increased length of inpatient stay, more hospital readmissions, and higher medication use, as well as raised burden and management costs (27–29). Therefore, one of the conclusions and agreements reached at this expert consensus meeting was recognizing the need of improving interaction, collaborative work, discussion, and agreements among experts involved in the management of patients with episodes of psychomotor agitation in order to share clinical experiences, and address and solve unmet needs. Furthermore, participants acknowledged the opportunity to discuss with colleagues from other settings and countries the experiences, difficulties, barriers, as well as commonalities and disparities in the different approaches in daily practice for managing episodes of psychomotor agitation in various clinical settings. It was also an opportunity to discuss local and international guidelines, and best clinical practice recommendations available locally or internationally to aid in the management of the agitated patient (2, 14–17, 21, 30).

**Figure 4:**
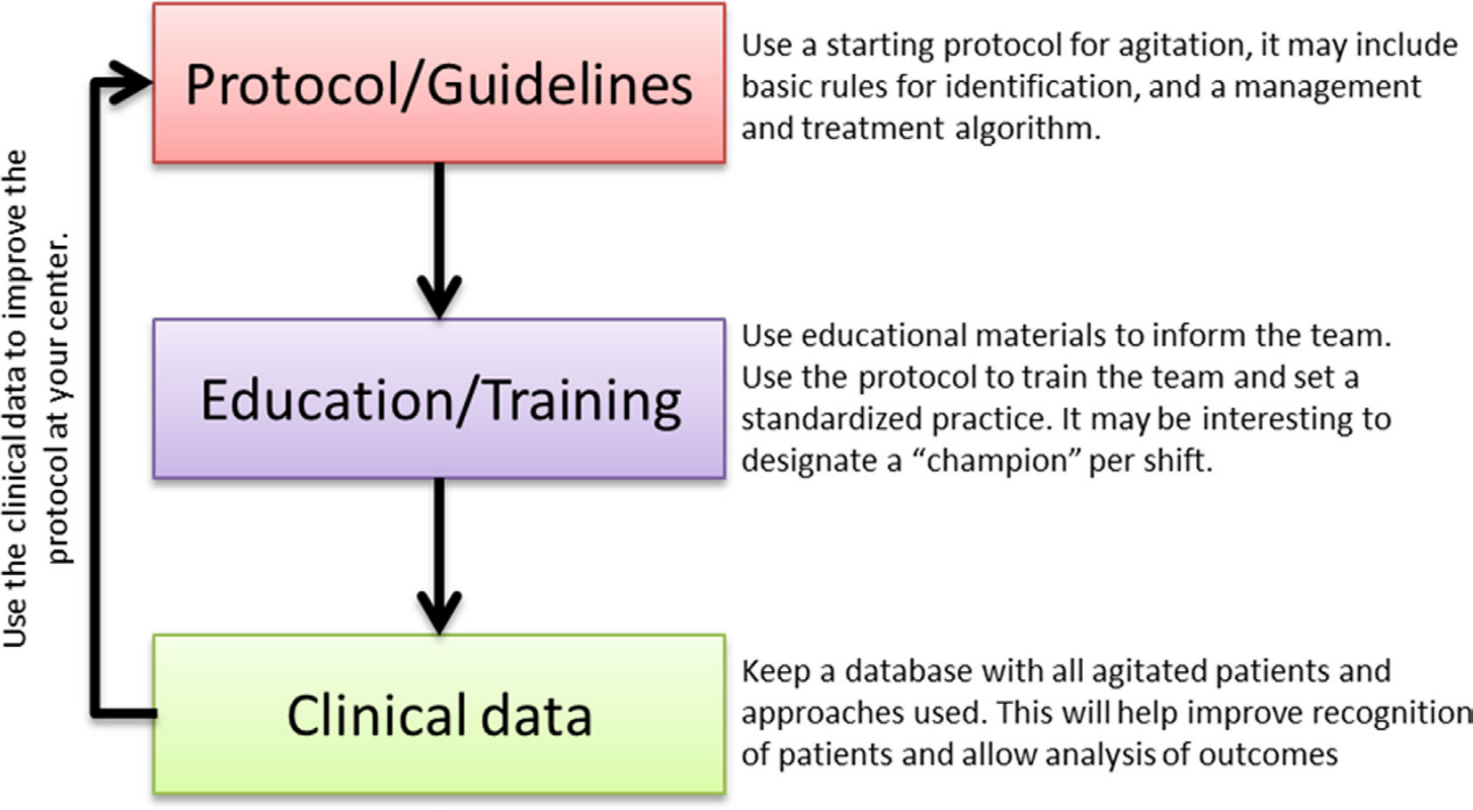
Action plan to reduce current barriers and optimize the procedures and interventions for managing the agitated patient.

In daily clinical practice, the three aspects that were discussed by the expert group, namely early identification, adequate management, and pharmacological treatment of agitation, are tightly intertwined. Thus, improvement of one of these areas may have a positive impact on the other ones. The potential for agitation to escalate into aggressive behavior, putting patients, staff, and others at risk, highlights the importance to address the behavior early, rapidly, and efficiently relying on the most adequate and effective pharmacological and non-pharmacological interventions for each patient to ensure the safety of everyone involved (6, 31). At the meeting, some solutions were identified by the expert participants to reduce the current barriers and, consequently, optimize the procedures and interventions for managing the agitated patient. However, such potential solutions did not target directly these three aspects of agitation but rather proposed more practical solutions that could be implemented in the day-to-day practice. An action plan is presented here (Figure 4) based on the discussion of the solutions at the meeting.

During the meeting, the workgroup agreed that it would be highly recommendable for the appropriate management of agitation to rely on standardized protocols at each center, particularly so, as treatment protocols and guidelines are commonly regarded as gold standards of care. In this context, an example of the updated treatment algorithm from the Hospital Clinic of Barcelona was presented and discussed (32). Such algorithm may be used as a starting point and adapted to each specific center. This would help the different members of multidisciplinary teams involved in the assessment and management of agitated patients to have a clearer understanding of the procedures to follow. Having standardized protocols would also help educate, train, and engage non-specialized and new staff in the early identification and management of the agitated patient. Since any protocol would have multiple decision choices, it is very important to keep track of the different procedures, so that the efficiency of each one can be measured. This would also provide evidence to guide any needed amendment to the original protocol.

The following strategic triad is highly relevant to advance the understanding of the most adequate management of agitation. The relevance lies on the continued improvement of the working flow: standardized protocols will improve an adequate assessment process, including methods of identification, management, and treatment of agitation. This will improve educational and training tools that will make a more efficient individual and team work possible. Standard procedures and trained professionals would allow an accurate registry of the agitation cases and analysis of the optimal actions. And closing the circle, this analysis will help improving the current protocols and algorithms, making possible a working method design that will allow updated optimal care of the agitated patient. This working plan requires some time and effort but may provide great improvements in the experience for patients, caregivers, and HCPs in the management of an episode of agitation, as well as a more efficient use of the economic and personal resources at each individual center.

## Author Contributions

JM-R moderated the meeting and overviewed the writing of the manuscript. All the authors contributed significantly to the discussion, ideas and conclusions presented here and reviewed critically, and approved the manuscript.

## Conflict of Interest Statement

GDS has received honoraria (speaker/advisory board) from Angelini, Otsuka, Janssen; RF has been speaker for some workshops/seminars for Lilly, Otsuka, Lundbeck, Janssen, and Bristol-Myers-Squibb; MG has received funding for research projects and/or honoraria as a consultant or speaker for Ferrer, Janssen, Lundbeck and Instituto de Salud Carlos III; GG has received honoraria from Laboratories Bago, Otsuka, Laboratories Baliarda, Roemers, Casasco, Ferrer, Bayer, Baguete Engineering in Anesthesiology and Pfizer; KGK received speaker honoraria by Eli Lilly, neuraxpharm, Servier and Trommsdorf; JK has received financial support for IIT studies from Medtronic EuropeSARL (Meerbusch, Germany) and he has occasionally received honoraria from Lundbeck, Otsuka, Servier, Janssen, Trommsdorff, and Schwabe for lecturing at conferences; KP has received honoraria from AOP Orphan, Eli Lilly, and Ferrer; FR from Ferrer for the work under consideration for publication (non-financial support); AR has received financial compensation from Ferrer, Lundbeck, Janssen-Cilag S.A., Lilly, Pfizer, and Otsuka and has collaborated with Elan Corporation and Cephalon Inc.; GS has received honoraria as a consultant or speaker for Ferrer, Janssen, Lundbeck, and Adamed; JS has received travel grants and speakers honoraria from AOP Orphan, Janssen, G.L.Pharma, and Lundbeck. The rest of the authors have declared no conflicts of interest.
